# Using a modified nominal group technique to develop complex interventions for a randomised controlled trial in children with symptomatic pes planus

**DOI:** 10.1186/s13063-022-06251-7

**Published:** 2022-04-11

**Authors:** Michael R. Backhouse, Daniel J. Parker, Stewart C. Morison, Jenny Anderson, Sarah Cockayne, Joy A. Adamson

**Affiliations:** 1 Warwick Clinical Trials Unit, Warwick Medical School, University of Warwick, Coventry, UK; 2 School of Health and Society, University of Salford, Salford, UK; 3 School of Life Course & Population Sciences, Kings College London, London, UK; 4 York Trials Unit, Department of Health Sciences, University of York, York, UK

**Keywords:** Consensus, Consensus development, Nominal group technique, Complex interventions, Foot health advice, Insoles, Orthoses, Orthotic devices, Exercise, Pes planus

## Abstract

**Background:**

Children with symptomatic flat feet (pes planus) frequently present for care but there remains uncertainty about how best to manage their condition. There is considerable variation in practice between and within professions. We intend to conduct a three-arm trial to evaluate three frequently used interventions for pes planus (exercise and advice, exercise and advice plus prefabricated orthoses, and exercise and advice plus custom made orthoses). Each of these interventions are complex and required developing prior to starting the trial. This paper focusses on the development process undertaken to develop the interventions.

**Methods:**

We used a modified Nominal Group Technique combining an electronic survey with two face-to-face meetings to achieve consensus on the final logic model and menu of options for each intervention. Using the Nominal Group Technique across consecutive meetings in combination with a questionnaire is novel, and enabled us to develop complex interventions that reflect contemporary clinical practice.

**Results:**

In total 16 healthcare professionals took part in the consensus. These consisted of 11 podiatrists, two orthotists, two physiotherapists, and one orthopaedic surgeon.

Both meetings endorsed the logic model with amendments to reflect the wider psychosocial impact of pes planus and its treatment, as well as the increasing use of shared decision making in practice. Short lists of options were agreed for prefabricated and custom made orthoses, structures to target in stretching and strengthening exercises, and elements of health education and advice.

**Conclusions:**

Our novel modification of the nominal group technique produced a coherent logic model and shortlist of options for each of the interventions that explicitly enable adaptability. We formed a consensus on the range of what is permissible within each intervention so that their integrity is kept intact and they can be adapted and pragmatically applied. The process of combining survey data with face-to-face meetings has ensured the interventions mirror contemporary practice and may provide a template for other trials.

**Supplementary Information:**

The online version contains supplementary material available at 10.1186/s13063-022-06251-7.

## Background

As children grow, most develop a medial longitudinal arch in their feet. In the early years, children’s feet are flat and flexible structures (known as pes planus), but most change over time with the emergence of a medial arch [1, 2]. Flat feet are therefore considered part of a child’s typical developmental trajectory and are usually asymptomatic [3, 4]. However, a substantial number of children develop symptoms that can be associated with their foot posture and there is emerging data on the impact this can have on their quality of life [5]. In addition to foot and ankle pain, children often report pain elsewhere in their legs and lower back, tiredness in their legs and struggle to walk the same distance as their peers; all of which can lead to reduced engagement with physical and childhood activities [6]. Effective management of symptomatic pes planus is therefore essential to keep children active and support their healthy physical, social and psychological development [7].

Children with symptomatic pes planus are treated by a range of professions including orthotists, physiotherapists, podiatrists, and surgeons [8]. Although there is an international consensus that symptomatic pes planus should be treated, there has long been debate about how they should be managed, or whether symptoms will resolve without intervention [9–11]. Currently, management of symptomatic pes planus varies considerably within and between professions [8]. Corrective surgery remains rare for flexible symptomatic pes planus but exercise, foot orthoses, and advice regarding suitable footwear are commonly provided in line with the international consensus [9, 12]. Repeated systematic reviews have highlighted the lack of primary evidence evaluating therapies and consistently emphasise the need for robust trials to evaluate interventions in the treatment of symptomatic paediatric pes planus [12–15].

Our trial *O*rthotic*s* for *Tr*eatment of Symptomatic Flat Feet *i*n *Ch*ildren (OSTRICH) was commissioned by the National Institute for Health Research – Health Technology Assessment Programme. It is designed to be a pragmatic comparison of the clinical and cost-effectiveness of three common treatments: Exercises and advice (control) compared to exercise and advice plus prefabricated orthoses (intervention); and exercise and advice plus custom made orthoses (intervention) (ISRCTN: 14602568). In the context of symptomatic pes planus, advice, exercises, and orthoses all contain multiple interacting components that need tailoring to meet the needs of individual patients. They can therefore be defined as complex interventions [15, 16].

Complex interventions consisting of multiple treatments are particularly difficult to standardise within trials, and then to subsequently implement in practice [16, 17]. However, attempting to standardise an intervention to meet the needs of researchers can lead to compromised outcomes [16–19], as artificially uniform interventions do not reflect how the interventions will be implemented in future clinical practice. Instead, a degree of flexibility is recommended to ensure that effective interventions can subsequently be adapted to local circumstances and patient need after the trial [16]. A previously advocated pragmatic solution is to standardise ‘function’ rather than ‘form’ [16, 17]. We therefore decided to produce a shortlist or menu of options for each treatment group in the OSTRICH trial rather than a single model/design of prefabricated/custom made orthoses, or list of exercises. This builds on a similar approach we have used in previous wound care trials [20].

Guidelines recommend modelling how interventions work prior to testing in order to understand the components of an intervention and their interrelationships [21]. Logic models are widely used for this purpose but these need to be developed and refined through formal methods to combine the opinion of clinical experts and the available literature [21, 22]. It has been argued that logic models should not be used in a way to forge a consensus that produces concise guidance that may be inappropriate across different settings, but instead, use the model to say what range is permissible [22]. Given the variation in how children with symptomatic pes planus are treated, we anticipated that clinicians would hold diverse opinions on the suitability of different interventions, and their mechanisms of action. We therefore decided to use a formalised consensus technique in the form of a modified nominal group technique (NGT), to refine our logic models and develop the short lists of suitable interventions. The NGT is a well-established, structured group facilitation technique that generates and prioritises responses to a given question by a group of people with expertise on the topic [23]. The main advantage of the NGT over other consensus techniques is that it enables live discussion of topics, and the opportunity for robust generation of ideas which was particularly pertinent for the logic models [23, 24].

The aims of the consensus meetings were to agree a logic model describing the mode of action for each of the interventions; and agree a shortlist or ‘menu’ of options for each intervention.

## Methods

We combined an electronic survey with two consensus development meetings which were facilitated using a modified nominal group technique to achieve consensus on the final logic model and menu of options for each intervention (Table 1).

**Table 1:** Overview of consensus development process

Time point	Activity
Prior to 1^st^ meeting	• Conduct survey • Develop version 1 of the logic model and menus
1st consensus development meeting	• Present version 1 of the logic model and menus at the first part of the meeting • Use NGT process to collate feedback on version 1 of the logic model and menus and develop version 2.
Between meetings	• Review version 2 of the logic model and menu for linguistic and structural clarity only
2nd consensus development meeting	• Present version 2 of the logic model and menus at the first part of the meeting • Use NGT process to collate feedback on version 2 of the logic model and menus and produce version 3 the final consensus

The first modification from the standard NGT design was that we used an electronic survey to generate items for subsequent discussion rather than doing this solely within the physical meeting. To do this we developed an online self-administered, survey using JISC Online Surveys (Bristol, UK) which we distributed to the named local Principle Investigator (PI) in each of the sites participating in the OSTRICH trial. The participating sites represent a geographical spread across England & Wales. Consent was implied by completion of the survey and it was accessible from 13/01/2020 to 31/01/2020. Questions were developed by experienced clinicians and subject experts from the OSTRICH trial team. Each site was asked to provide one response to each question. The survey consisted of 12 questions to describe the clinical experience of responders and details of the clinical treatments they provided for children with symptomatic pes planus (Additional file 1). In the absence of universally agreed definitions of different types of orthosis, we standardised definitions used across this study and the main OSTRICH trial (Table 2). These were in line with previous international surveys of orthoses prescription habits [25, 26].

**Table 2:** Definitions of different types of foot orthoses

Categories were defined to align with previous international surveys of orthoses prescription habits [25, 26]	
- **Simple orthoses** Flat orthoses with or without padding to accommodate painful areas or lesions. - **Prefabricated orthoses** Devices made to a generic foot shape, contoured for the arch, and included modular prefabricated orthoses that can be altered by clinicians (e.g. by the addition of heel posting, wedges, pads or top covers). - **Custom made orthoses** Manufactured for a specific person based on a 3D impression or computerised image of that person’s foot, and produced using computer-aided device/manufacturing (CAD/CAM) or more traditional manufacturing techniques (e.g. foam impression box or plaster of Paris cast).	

At the same time, we designed the initial version of the logic model to be discussed at the first consensus meeting. This was developed using the expertise of clinicians on the OSTRICH research team and available literature that described the mechanism of action of each of the interventions. We developed a Type 3 logic model as these focus on the interventions rather than the setting in which they are implemented; include a precise list of intervention components; and provide a clear sense of how interventions lead to outcomes [22].

We invited clinicians from sites intending to participate in the OSTRICH trial to attend one of the meetings and encouraged a mixture of professions who are routinely involved in the management of symptomatic pes planus. We held two iterative consensus development meetings in different locations (University of Brighton and University of Salford) to enable as many clinicians to attend as possible.

Each meeting followed a predetermined agenda including introductions to each other, an overview of the planned OSTRICH trial, and an explanation of how the nominal group technique (NGT) was to be employed. Feedback was also sought on the clarity of eligibility criteria for the main trial, definitions of the orthoses groups, and how adherence was measured in clinical practice. The meetings were facilitated by members of the OSTRICH team (JA, JAA, MB, SM, and DP) and discussions were captured through audio recordings and written field notes (DP, JA) to ensure key points were accurately captured. The meetings were intended to be a welcoming safe space for participants to speak openly about their practice. Following an explanation of how the meeting would work, the facilitator asked the group the two nominal questions:

i) What is the mechanism of action for each of the three categories of intervention?
ii) Which specific prefabricated orthoses, custom made orthoses, and exercises and advice are suitable to go on the menu of acceptable options.

To enable the group to answer the first question, the facilitator introduced logic models, definitions of key terms in the model, and talked the participants through each step of the model. At the first meeting, we presented the initial logic model and sought suggestions to improve it. Suggestions from each group were then discussed, modified if required, and either accepted or rejected by the group. This process was repeated until no further suggestions were made by participants, and an agreement was reached. Participants at the second meeting used the same process whereby they reviewed and amended the model created at the first meeting, which enabled the production of the final model.

We then shared information on the different orthoses identified through the questionnaire to help the group answer the second nominal question. We presented the following information on each orthosis: make; model; a description of the shape; details of which materials were used for the shell/heel cup midlayer and topcover; details of any additional features (such as additional posting options); and which sizes they came in. We also provided an image of each model. Participants were encouraged to discuss each model, share their experiences of using it, and their thoughts on its suitability for the trial population. Participants were also encouraged to suggest additional models that were not included in the survey responses and these were discussed by the group in the same way. After each model had been discussed, the group used the online voting platform Mentimeter (Mentimeter AB, Stockholm, Sweden) to anonymously vote on the suitability of each model. This further evolution of the standard NGT process maintained the anonymity of voting described in the original NGT and facilitated administration. Participants had the option to vote whether each orthosis was ‘suitable’ or ‘not suitable’, or they could abstain. We determined that at least three members of the panel would need to agree on the suitability of an intervention for it to be included. With no universally agreed level of consensus in the literature we chose this level to emphasise the pragmatic nature of our intervention in the subsequent trial. This process was repeated at the second meeting to produce the final list.

The same iterative process was then followed for custom made orthoses, and to determine which modifications were acceptable to the orthoses. Then, we conducted the process on exercises to determine which structures should be targeted, and what information should be contained in the advice intervention.

## Results

Sixteen healthcare professionals took part in the consensus meetings (7 in Eastbourne, and 9 in Salford). These consisted of 11 podiatrists, two orthotists, two physiotherapists, and one orthopaedic surgeon.

**Fig. 1:**
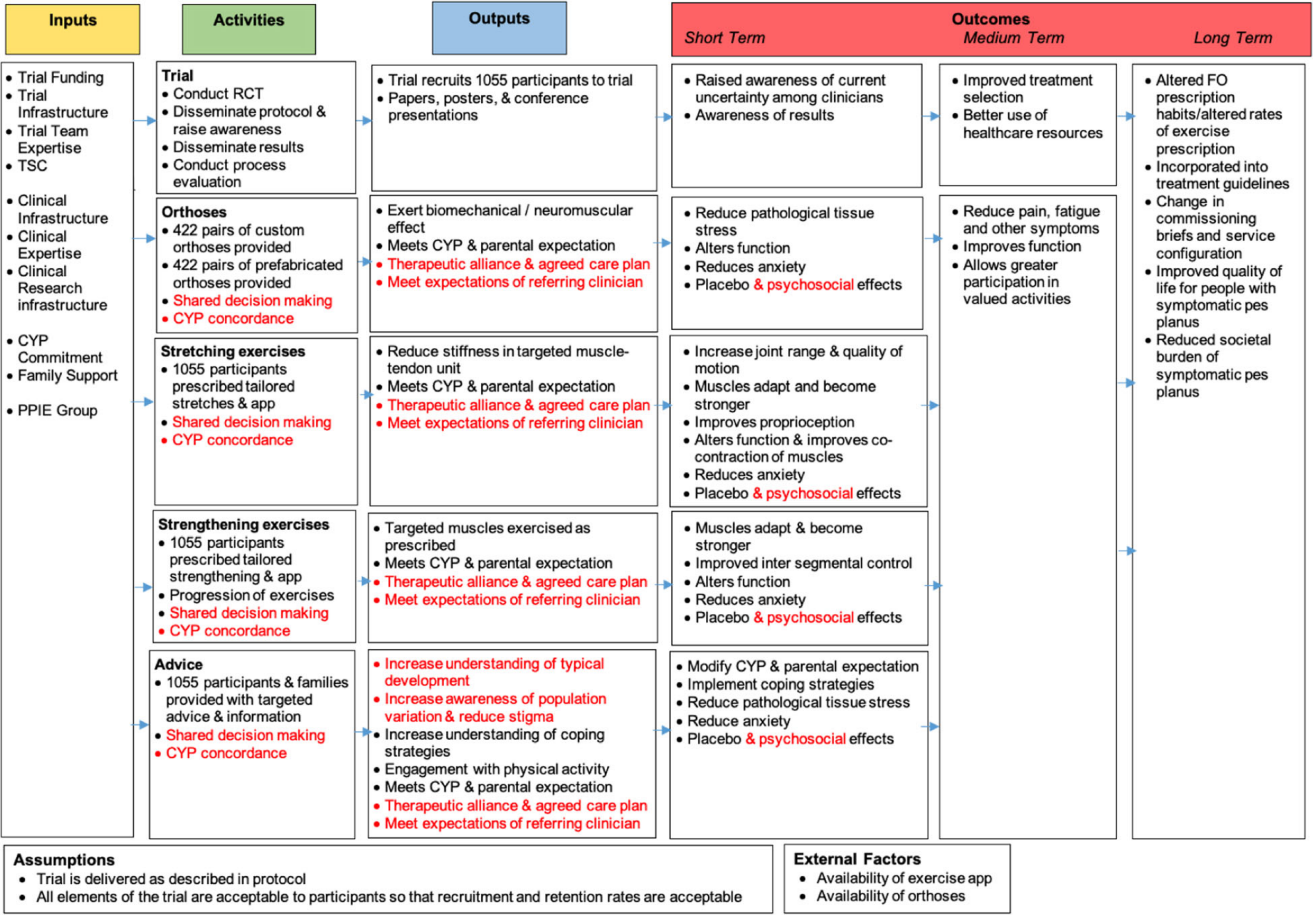
Logic model. Changes made from the original model during the consensus meetings are shown in red. TSC, Trial Steering Committee; CYP, child or young person; PPIE Group, Patient and Public Involvement and Engagement Group; RCT, randomised controlled trial; FO, foot orthoses

Both meetings endorsed the logic model and the only amendments suggested were to reflect the wider psychosocial impact of pes planus and its treatment, as well as the increasing use of shared decision making in practice. As a result of this, terminology such as ‘adherence’ was changed to ‘concordance’ and the outputs and outcomes were amended to better reflect the psychosocial aspects. The final logic model is shown in Fig. 1.

The number of votes for each model of prefabricated orthoses are presented in Table 3. Six additional models were suggested during the nominal group process at the second meeting so could not be voted upon at the first meeting. Five of the suggested orthoses received one or less votes so were excluded as there was no consensus on their suitability.

**Table 3:** Prefabricated foot orthoses

	Eastbourne	Salford	Sum
Kent orthotic	6	8	14
Algeos - Kids feet in motion	6	6	12
Slimflex - Simples	4	8	12
Formthotics - Junior	5	6	11
Healthy Step - Pedipod	5	6	11
Interpods - Flex	5	5	10
Interpods - Soft	2	8	10
Peapods - Dinky	3	7	10
Peapods - Junior	4	5	9
Algeos - Kiddy-thotics	3	4	7
Slimflex - Amber	2	5	7
Slimflex - Green	3	4	7
Talarmade - Kidzstep	3	4	7
Talarmade - Prostep	4	3	7
Slimflex - Carbon		7	7
Talarmade - 4kids	3	3	6
Xline - 70		6	6
Xline - RF		6	6
Haplabase	1	4	5
LBG - Biomex	3	2	5
Vectorthotics		5	5
Xline - Standard		5	5
Powersteps - Powerkids	2	2	4
Talarmade -Basis pros		3	3
Silipos - Achilles heel pad	0	1	1
Talarmade - 1st line	0	1	1
Talarmade – Elite	0	1	1
Algeos - S-gel heel cup	0	0	0
Wonderspur - Soft heel cup	0	0	0

Slimflex – carbon, Xline – 70, Xline – RF, Vectorthotics, and Talarmade – Basis Pros were added at the Salford meeting so could not be scored at the Eastbourne meeting. Numbers reflect the number of participants voting that each option was suitable to be added to the menu of acceptable options

Custom made orthoses are more complex than prefabricated orthoses, in that the practitioner can select a combination of materials for the orthoses. At both meetings, there was in-depth discussion of both the different materials that could be used, as well as how to categorise them. The categorisation of materials evolved from how it was presented in the initial questionnaire (Additional file 1), through the first meeting, and to the final categorisation. Thus, the data in Table 4 reflects the final categories agreed at the Salford meeting. In contrast, there was little variation in how the additions and modifications were described as shown in Table 5. All of the additions and modifications were voted as being suitable except the gait plate extension.

**Table 4:** Custom made orthoses

Options	Votes
**Shell material**	
Single material - Rigid (does not bend)	7
Single material -Semi rigid (some flexibility)	8
Single material -Flexible/cushioning (does bend/cushions)	3
Multi density (Rigid)	7
Multi density (semi-rigid)	8
Multi density (flexible/cushioning)	3
**Posting**	
None	6
Intrinsic posting	7
Extrinsic posting	7
**Top cover material**	
None	7
Minimal (e.g. leather/vinyl)	8
Cushioning (e.g. Poron or similar polyurethane)	7
Cushioning with modifications to offload specific areas	7

Numbers reflect the number of participants voting that each option was suitable to be added to the menu of acceptable options

**Table 5:** Additions and modifications to orthoses

	Eastbourne	Salford	Sum
Plantar groove	5	7	12
1st ray cut out	5	8	13
Arch height/filler/reinforce	5	8	13
Heel cup	5	8	13
Heel raise	5	8	13
Medial extrinsic rear foot post	5		5
1st met cutout	5	7	12
Navicular dell	3	7	10
Lateral heel skive	3	7	10
Medial heel skive	3	7	10
Kinnetic wedge – Dannenberg Device	3	7	10
Lateral clip	1	5	6
Lateral flange	1	7	8
Heel extrinsic post – lateral flare,	1	8	9
Gait plate extension	0		0

Mentimeter voting information was not captured for ‘Medial extrinsic rearfoot post’, and ‘gait plate extension’ at the Salford meeting. Numbers reflect the number of participants voting that each option was suitable to be added to the menu of acceptable options

Results of the voting on the anatomical structures that could be targeted with stretches and strengthening exercises are presented in Table 6. The majority of these were deemed suitable for stretching in the target population with the exception of stretching the tibialis posterior muscle and the arch. Similarly, the vast majority of structures were considered suitable targets for strengthening with the exception of the plantar fascia and arch.

**Table 6:** Exercises

Target Structure	Eastbourne	Salford	Sum
**Stretching exercises**			
Calf (gastrocnemius/soleus/achilles)	6	9	15
Hamstrings	6	9	15
Quadriceps	6	8	14
Iliotibial band	5	7	12
Plantar fascia	4	7	11
Peroneals	4	6	10
Gluteals	3	7	10
Intrinsic foot muscles	2	5	7
Long flexors (FHL, FDL)	1	6	7
Tibialis posterior	1	1	2
Arch	0	0	0
**Strengthening exercises**			
Calf (gastrocnemius/soleus/achilles)	6	9	15
Long flexors (inc. tibialis posterior)	6	9	15
Core Muscles	6	9	15
Quadriceps	6	9	15
Intrinsic foot muscles	5	9	14
Gluteals	5	9	14
Hamstrings	5	9	14
Peroneals	5	8	13
Plantar fascia	0	0	0
Arch	0	0	0
**Other Exercises**			
Games and play	6	8	14
Balance exercises	5	8	13
Plyometrics	3	7	10
Activity prescription	6		6

Mentimeter voting information was not captured for ‘Activity Prescription’ at the Salford meeting. Numbers reflect the number of participants voting that each option was suitable to be added to the menu of acceptable options

All of the topics listed in Table 7 were deemed suitable areas of education and advice for inclusion in the menu of options for the full trial.

**Table 7:** Education and advice

Content	Eastbourne	Salford	Sum
Normal and abnormal morphological variation in a population	6	9	15
Normal arch development in children	6	9	15
Suitable footwear	6	9	15
Structures around the symptomatic area (including muscles & bones)	6	9	15
How to maintain participation/activity etc	6	9	15
Public health messages (e.g. healthy weight, exercise, heart health, wellbeing)	6	9	15
The long term effects—changes expected with age	6	8	14
Muscle and bone growth	5	9	14
Relationship between gastroc tightness and painful pes planus	5	9	14
Demographic/global differences	4	9	13
Symptoms related to balance/trips and falls		8	8
Sex differences		7	7

Symptoms related to balance/trips and falls, and sex differences were added at the Salford meeting so could not be voted upon at the Eastbourne meeting. Numbers reflect the number of participants voting that each option was suitable to be added to the menu of acceptable options

## Discussion

Our use of a modified NGT process enabled us to produce a logic model and menu of options for each of the complex interventions we intend to test within the OSTRICH trial. Although the NGT is a well-established group facilitation technique [23], using it across consecutive meetings in combination with a questionnaire and electronic voting is novel, and enabled us to develop complex interventions that reflect contemporary clinical practice.

The increasing interest in complex interventions within the literature is in part due to the growing recognition that most interventions in healthcare are to some degree complex and the methodological challenges this represents [16, 27]. How these complex interventions are developed for trials has been the subject of considerable attention, but there is a recognition of the need to focus on developing techniques that produce interventions which can be adapted during their clinical implementation [22, 28]. Given how broad the spectrum of complex interventions used in healthcare is, it’s unlikely that a single technique would be suitable to develop interventions for all clinical situations. However, the development process we used is flexible and may provide a template for future trials.

The prefabricated orthoses we intend to use in our trial provide an excellent example of how complex interventions need to be adapted for implementation at different locations. NHS clinicians will not have access to each of the multiple models of prefabricated orthoses available on the market but will instead have a limited selection of devices within their Trust. Each device will be selected to fulfil a certain need and chosen based on cost and personal preference. Another clinician in a neighbouring trust may have an entirely different range of orthoses to choose from, but the range they have access to will have been chosen to meet the same clinical needs. Therefore, when developing our prefabricated orthoses intervention, it is important that we have built-in flexibility to enable implementation both within and beyond the trial.

Although our logic model may be of interest to future trials of foot orthoses, the development process we used could be of interest beyond foot and ankle research. Our logic model and menu of options for each intervention explicitly enable adaptability and provide a solution to an issue identified by Mills et al [22, 29] whereby sometimes models and interventions are too prescriptive to enable implementation. The process we used to develop them did not force consensus that produces precise guidance that is inappropriate across different settings and becomes redundant beyond the trial. Instead, we have formed consensus on the range of what is permissible within each intervention so that the integrity of the intervention is kept intact, yet they can be applied pragmatically.

The selections made at the two meetings reflect current practice and will support sites to deliver the trial and implementation of findings beyond that. Although there are a number of options for prefabricated orthoses, in particular, this reflects the breadth of options available on the market. How frequently each of these will be used in the OSTRICH trial remains to be seen, and we anticipate that a small number of prefabricated orthoses will be used in the vast majority of centres. The exact composition of this list would inevitably change if we preselected a different level of agreement for the votes within our meetings, or included different clinicians from different Trusts. It is though of interest, that the orthoses that were deemed unsuitable (i.e. were not voted as suitable) included devices that were only nominated by a single site in the survey prior to the first meeting. These included cushioning silicone heel pads and heel cups, which would not have been compatible with the mechanism of action described in the logic model. Perhaps the exclusion devices are therefore an expression of the internal validity and generalisability of the process.

Our process combined a survey to capture views from as many sites as possible, face-to-face discussions to enable detailed discussion, and an electronic voting method to provide a quantifiable measure of consensus. Utilisation of these complimentary aspects should be considered a strength of the study and may represent the ongoing evolution of the NGT process. We also consider it likely that they could be successfully transferred to an online format. We sought to make the two meetings multidisciplinary and although we achieved this, it is noticeable that the vast majority of attendees were podiatrists. Whether this is a strength or limitation, is unclear as our focus was on treatments which are predominantly provided by podiatrists. Future research could consider whether an alternative sampling strategy, more even distribution of professions and patients, and exploration of clinician experience would be beneficial.

## Conclusions

We describe a novel, modification of the nominal group technique that enabled us to produce a logic model and menu of options for each of the complex interventions we will test in the OSTRICH trial. By using the modified NGT technique participants were able to actively express their views in structured discussions, and then reach a final consensus. Whilst the consensus expressed by the group will be of interest to those conducting research in children with symptomatic pes planus, the template is adaptable and may be of use to a broader audience conducting research on complex interventions.

## Supplementary Information

**Additional file 1.**

## Data Availability

All data generated or analysed during this study are included in this published article and its supplementary material.

## Abbreviations

3D: Three dimensional
CAD/CAM: Computer-aided design/computer-aided manufacture
ISRCTN: International Standard Registered Clinical/social sTudy Number
NGT: Nominal group technique
NHS: National Health Service
OSTRICH: Orthotics for Treatment of Symptomatic Flat Feet in Children

## References

[1] Gijon-Nogueron G, Martinez-Nova A, Alfageme-Garcia P, Montes-Alguacil J, Evans AM (2019). International normative data for paediatric foot posture assessment: a cross-sectional investigation. BMJ Open.

[2] Volpon JB (1994). Footprint analysis during the growth period. J Pediatr Orthop.

[3] Martinez-Nova A (2018). Foot posture development in children aged 5 to11 years: A three-year prospective study. Gait Posture.

[4] Morrison SC, McClymont J, Price C, Nester C (2017). Time to revise our dialogue: how flat is the paediatric flatfoot?. J Foot Ankle Res.

[5] Kothari A, Stebbins J, Zavatsky AB, Theologis T (2014). Health-related quality of life in children with flexible flatfeet: a cross-sectional study. J Child Orthop.

[6] Roth-Isigkeit A, Thyen U, Stöven H, Schwarzenberger J, Schmucker P (2005). Pain among children and adolescents: restrictions in daily living and triggering factors. Pediatrics.

[7] Wrotniak BH, Epstein LH, Dorn JM, Jones KE, Kondilis VA (2006). The relationship between motor proficiency and physical activity in children. Pediatrics.

[8] Morrison SC, Tait M, Bong E, Kane KJ, Nester C (2020). Symptomatic pes planus in children: a synthesis of allied health professional practices. J Foot Ankle Res.

[9] Dars S, Uden H, Kumar S, Banwell HA (2018). When, why and how foot orthoses (FOs) should be prescribed for children with flexible pes planus: a Delphi survey of podiatrists. PeerJ.

[10] Evans AM (2008). The flat-footed child -- to treat or not to treat: what is the clinician to do?. J Am Podiatr Med Assoc.

[11] Harris EJ, Vanore JV, Thomas JL, Kravitz SR, Mendelson SA, Mendicino RW, Silvani SH, Gassen SC (2004). Diagnosis and treatment of pediatric flatfoot. J Foot Ankle Surg.

[12] Dars S, Uden H, Banwell HA, Kumar S (2018). The effectiveness of non-surgical intervention (Foot Orthoses) for paediatric flexible pes planus: A systematic review: Update. PLoS One.

[13] Rome K, Ashford RL, Evans A (2010). Non-surgical interventions for paediatric pes planus. Cochrane Database Syst Rev.

[14] Jane MacKenzie A, Rome K, Evans AM (2012). The efficacy of nonsurgical interventions for pediatric flexible flat foot: a critical review. J Pediatr Orthop.

[15] Evans AM, et al. Foot orthoses for treating paediatric flat feet. Cochrane Database Syst Rev. 2022;1(1). 10.1002/14651858.CD006311.pub4. 10.1002/14651858.CD006311.pub4 PMC8790962 35080267

[16] Datta J, Petticrew M (2013). Challenges to evaluating complex interventions: a content analysis of published papers. BMC Public Health.

[17] Hawe P, Shiell A, Riley T (2004). Complex interventions: how "out of control" can a randomised controlled trial be?. BMJ.

[18] Schroer S, Adamson J (2011). Acupuncture for depression: a critique of the evidence base. CNS Neurosci Ther.

[19] Council, M.R (2000). A framework for development and evaluation of RCTs for complex interventions to improve health.

[20] Ashby RL, Gabe R, Ali S, Adderley U, Bland JM, Cullum NA, Dumville JC, Iglesias CP, Kang'ombe AR, Soares MO, Stubbs NC, Torgerson DJ (2014). Clinical and cost-effectiveness of compression hosiery versus compression bandages in treatment of venous leg ulcers (Venous leg Ulcer Study IV, VenUS IV): a randomised controlled trial. Lancet.

[21] Moore GF, Audrey S, Barker M, Bond L, Bonell C, Hardeman W, Moore L, O'Cathain A, Tinati T, Wight D, Baird J (2015). Process evaluation of complex interventions: Medical Research Council guidance. BMJ.

[22] Mills T, Lawton R, Sheard L (2019). Advancing complexity science in healthcare research: the logic of logic models. BMC Med Res Methodol.

[23] Sondergaard E (2018). Using a modified nominal group technique to develop general practice. BMC Fam Pract.

[24] Gallagher M (1993). The nominal group technique: a research tool for general practice?. Fam Pract.

[25] Chapman LS, Redmond AC, Landorf KB, Rome K, Keenan AM, Waxman R, Alcacer-Pitarch B, Siddle HJ, Backhouse MR (2019). Foot orthoses for people with rheumatoid arthritis: a survey of prescription habits among podiatrists. J Foot Ankle Res.

[26] Chapman LS, Redmond AC, Landorf KB, Rome K, Keenan AM, Waxman R, Alcacer-Pitarch B, Siddle HJ, Backhouse MR (2018). A survey of foot orthoses prescription habits amongst podiatrists in the UK, Australia and New Zealand. J Foot Ankle Res.

[27] Craig P, Dieppe P, Macintyre S, Michie S, Nazareth I, Petticrew M (2008). Developing and evaluating complex interventions: the new Medical Research Council guidance. BMJ.

[28] Fletcher A, Jamal F, Moore G, Evans RE, Murphy S, Bonell C (2016). Realist complex intervention science: Applying realist principles across all phases of the Medical Research Council framework for developing and evaluating complex interventions. Evaluation (Lond).

[29] Baxter R, Murray J, O’Hara JK, Hewitt C, Richardson G, Cockayne S, Sheard L, Mills T, Lawton R, on behalf of the PACT research team (2020). Improving patient experience and safety at transitions of care through the Your Care Needs You (YCNY) intervention: a study protocol for a cluster randomised controlled feasibility trial. Pilot Feasibility Stud.

