# Correction: Using a modified nominal group technique to develop complex interventions for a randomised controlled trial in children with symptomatic pes planus

**DOI:** 10.1186/s13063-022-06324-7

**Published:** 2022-05-09

**Authors:** Michael R. Backhouse, Daniel J. Parker, Stewart C. Morrison, Jenny Anderson, Sarah Cockayne, Joy A. Adamson

**Affiliations:** 1 Warwick Clinical Trials Unit, Warwick Medical School, University of Warwick, Coventry, UK; 2 School of Health and Society, University of Salford, Salford, UK; 3 School of Life Course & Population Sciences, Kings College London, London, UK; 4 York Trials Unit, Department of Health Sciences, University of York, York, UK

**Correction to: Trials 23, 286 (2022)**

**https://doi.org/10.1186/s13063-022-06251-7**

Following the publication of the original article [1], we were notified that the third author name was spelled incorrectly as Stewart Morison instead of Stewart Morrison (double “r”).

The original article has been corrected.
