# Supplementary material for: Using a modified nominal group technique to develop complex interventions for a randomised controlled trial in children with symptomatic pes planus
Source: Trials. 2022 Apr 11;23:286. doi: 10.1186/s13063-022-06251-7 (PMC8996675; doi:10.1186/s13063-022-06251-7)
Supplement: Supplementary file 1 — Additional file 1. [file 13063_2022_6251_MOESM1_ESM.pdf]

OSTRICH Clinician Survey

Page 1: OSTRICH Clinician Survey

Thank you for your interest in this survey, which we are conducting to better understand key aspects of the treatments used in children with symptomatic pes planus. Results from this survey will help us to develop the treatment options provided in the OSTRICH Trial and inform the subsequent consensus meetings.

At the end of the study we will save some of the data for 20 years in case we need to check it. All aspects of this study comply with the General Data Protection Regulation and Data Protection Act. If you would like further information about how we use your information at https://www.york.ac.uk/healthsciences/research/trials/trials-gdpr/.

In this survey, the following definitions will be used:

Simple Foot Orthoses refer to flat insoles with or without padding to accommodate painful areas or lesions

Prefabricated Foot Orthoses refer to orthoses that are made to a generic foot shape. They are contoured for the arch and include modular prefabricated orthoses that can be altered by clinicians (e.g. by the addition of posting, wedges, pads, or top covers).

Custom made foot orthoses refer to orthoses that are manufactured for a specific person based on a 3D impression or computerised image of that person's foot. Custom FOs may be produced using CAD/CAM, or more traditional manufacturing techniques (e.g. foam impression box or plaster of Paris cast).

This survey has been reviewed and approved by the University of York, Department of Health Sciences Research Governance Committee.

If you have any questions or would like any further information, please contact Dr Mike Backhouse email mike.backhouse@york.ac.uk

Thank you for completing this survey.

Page 2: Information about you and your practice

1. Please select your profession

- Nurse
- Orthopaedic Surgeon
- Orthotist
- Physiotherapist
- Podiatrist
- Other

1.a. If you selected Other, please specify:

2. In which year did you qualify?

3. In which country do you conduct most of your clinical work?

- England
- Scotland
- Wales
- Northern Ireland
- Republic of Ireland
- Other

3.a. If you selected Other, please specify:

4. What proportion of your caseload do paediatric foot and ankle problems account for (%)?

5. On average, how many children with symptomatic pes planus do you see in clinic each month?

Page 3: Information about the treatments you provide to children with pes planus

Simple Foot Orthoses refer to flat insoles with or without padding to accommodate painful areas or lesions

Prefabricated Foot Orthoses refer to orthoses that are made to a generic foot shape. They are contoured for the arch and include modular prefabricated orthoses that can be altered by clinicians (e.g. by the addition of posting, wedges, pads, or top covers).

Custom made foot orthoses refer to orthoses that are manufactured for a specific person based on a 3D impression or computerised image of that person's foot. Custom FOs may be produced using CAD/CAM, or more traditional manufacturing techniques (e.g. foam impression box or plaster of Paris cast).

6. Which of the following treatments do you prescribe for children and young people with symptomatic pes planus? (select all that apply)

- ☐ I do not prescribe any treatment for this condition
- ☐ I do not prescribe specific treatments for this condition, but refer patients to colleagues who do
- ☐ Advice and education
- ☐ Braces
- ☐ Custom made foot orthoses
- ☐ Exercises aiming to stretch specific soft tissue structures
- ☐ Exercises aiming to strengthen specific soft tissue structures
- ☐ Prefabricated foot orthoses
- ☐ Simple foot orthoses
- ☐ Specialist footwear
- ☐ Strapping / Taping
- ☐ Surgery
- ☐ Other

6.a. If you selected Other, please specify:

7. Which of the following treatments do you **most commonly prescribe as first line therapy** for children and young people with symptomatic pes planus? (if you typically prescribe multiple therapies in a package, please select all options which apply)

- ☐ I do not prescribe any treatment for this condition
- ☐ I do not prescribe specific treatments for this condition, but refer patients to colleagues who do
- ☐ Advice and education
- ☐ Braces
- ☐ Custom made foot orthoses
- ☐ Exercises aiming to stretch specific soft tissue structures
- ☐ Exercises aiming to strengthen specific soft tissue structures
- ☐ Prefabricated foot orthoses
- ☐ Simple foot orthoses
- ☐ Specialist footwear
- ☐ Strapping / Taping
- ☐ Surgery
- ☐ Other

7.a. If you selected Other, please specify:

8. If you provide advice and education to children with symptomatic pes planus, which of the following would it typically contain? (tick all that apply)

- ☐ I do not provide advice and education
- ☐ Normal & abnormal morphological variation in a population
- ☐ Normal arch development in children
- ☐ Suitable footwear
- ☐ Other

8.a. If you selected Other, please specify:

9. If you prescribe exercises to **stretch** specific soft tissue structures please indicate which structures you typically target (select all that apply)

- ☐ I do not prescribe exercises to stretch soft tissue structures
- ☐ I refer to another professional to provide specific exercises
- ☐ I prescribe exercises to stretch specific soft tissue structures

9.a. Which structures do you typically target?

10. If you prescribe exercises to **strengthen** specific soft tissue structures please indicate which structures you target? (select all that apply)

- [ ] I do not prescribe exercises to strengthen soft tissue structures
- [ ] I refer to another professional to provide specific exercises
- [ ] I prescribe exercises to strengthen specific soft tissue structures

10.a. Which structures do you typically target?

11. If you prescribe prefabricated foot orthoses for children with pes planus, which brand and model do you typically use?

- ○ I do not prescribe prefabricated foot orthoses
- ○ I refer to another professional to provide foot orthoses
- ○ I prescribe prefabricated foot orthoses for children with pes planus

11.a. Which brand and model of prefabricated foot orthoses do you typically use?

12. If you prescribe custom made foot orthoses for children with pes planus, which type of material do you use most frequently for the following components:

- ○ I do not prescribe custom made foot orthoses
- ○ I refer to another professional to provide foot orthoses
- ○ I prescribe custom made foot orthoses for children with pes planus

12.a. Which type of material do you use most frequently for the **shell**?

- ☐ Highly rigid (e.g. carbon fibre)
- ☐ Semi rigid (e.g. polypropylene)
- ☐ Semi flexible (e.g. high density EVA)
- ☐ Highly flexible / cushioning (e.g. medium or low density EVA)

12.b. Which type of material do you use most frequently for the **rearfoot posting**?

- ☐ None
- ☐ Intrinsic posting
- ☐ Highly rigid (e.g. carbon fibre)
- ☐ Semi rigid (e.g. polypropylene)
- ☐ Semi flexible (e.g. high density EVA)
- ☐ Highly flexible / cushioning (e.g. medium or low density EVA)

12.c. Which type of material do you use most frequently for the **top cover**?

- ☐ Minimal (e.g. leather / vinyl)
- ☐ Cushioning (e.g. Poron or similar polyurethane)
- ☐ Cushioning with modifications to offload specific areas

13. Thank you for completing this survey, if you would like to make any additional comments please do so here.

Page 4

Thank you for completing this questionnaire - your answers are important in helping to determine the interventions we use in OSTRICH.

If you have any further questions or comments, please contact Dr. Mike Backhouse on mike.backhouse@york.ac.uk.

UNIVERSITY of York
